# Supplementary material for: Optical control of polarization in ferroelectric heterostructures
Source: Nat Commun. 2018 Aug 21;9:3344. doi: 10.1038/s41467-018-05640-4 (PMC6104049; doi:10.1038/s41467-018-05640-4)
Supplement: Supplementary file 1 — Supplementary Information [file 41467_2018_5640_MOESM1_ESM.pdf]

Supplementary Information

Optical Control of Polarization in Ferroelectric Heterostructures

Tao Li, Alexey Lipatov, Haidong Lu, Hyungwoo Lee, Jung-Woo Lee, Engin Torun, Ludger Wirtz, Chang-Beom Eom, Jorge Iniguez, Alexander Sinitskii, and Alexei Gruverman

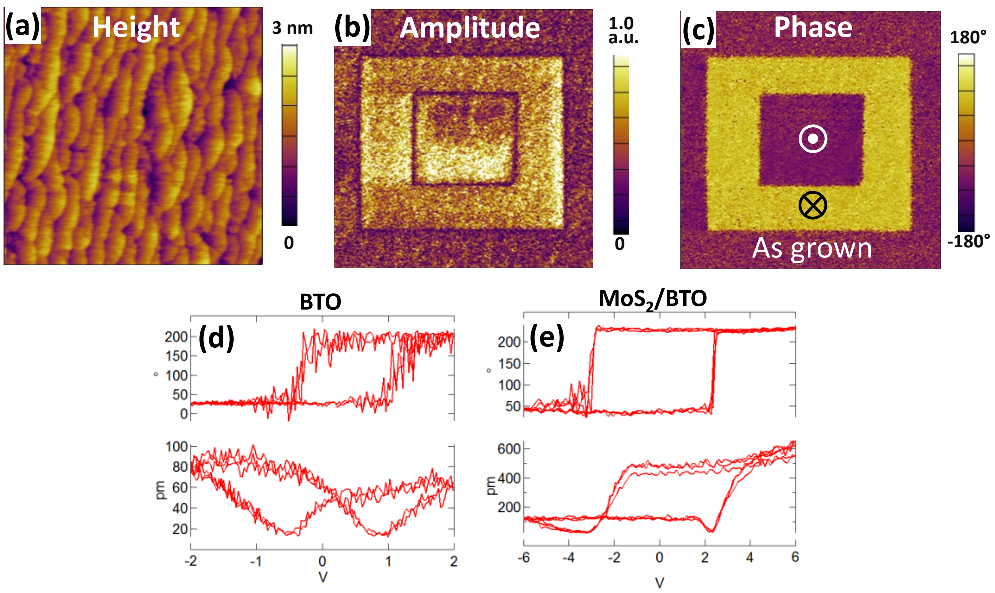

Supplementary Figure 1. **PFM characterization of the as-grown $BaTiO_3$ thin films**. (a) Topographic image of epitaxial 12-u.c.-thick $BaTiO_3$ (BTO) film. (b,c) PFM amplitude (b) and PFM phase (c) images of the BTO film with inner (outer) square regions electrically poled to the upward (downward) polarization by scanning the regions with -3V (+3V) bias applied to the conductive probe. (d,e) PFM hysteresis loops acquired on the as-grown BTO film (d) and on MoS_2/BTO/SRO heterostructure (e).

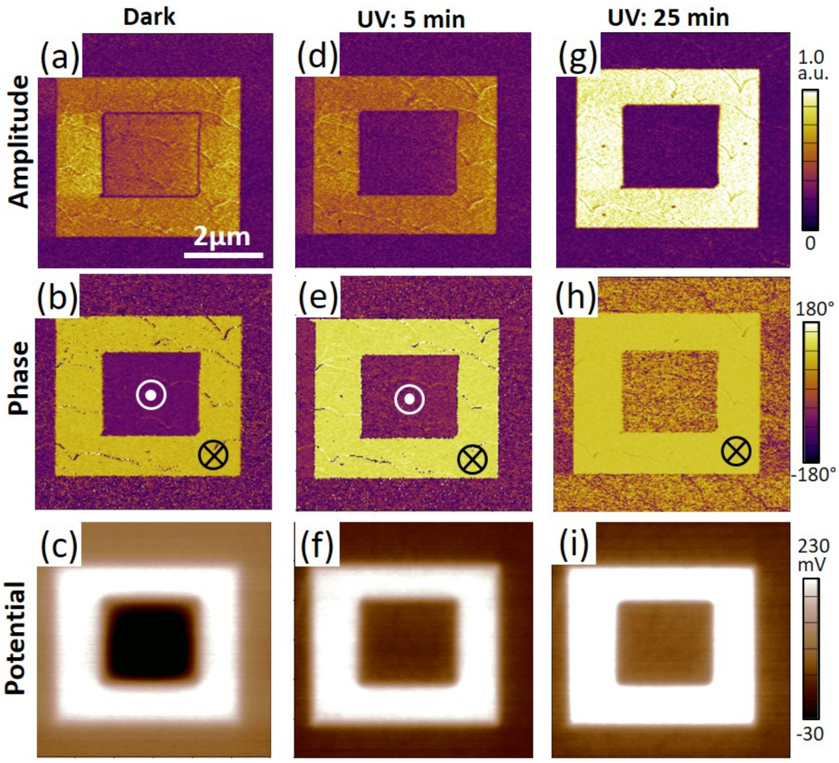

Supplementary Figure 2. Response of the bare $BaTiO_3$ film to UV illumination. PFM amplitude (a, d, and g) and phase (b, e, and h) images show a gradually reducing amplitude for the P_{up} domain and an increasing amplitude for the P_{down} domain upon UV illumination, which illustrates the polarization-dependent redistribution of the photoinduced charges. However, no polarization reversal was observed. (c, f, i) Concomitantly, KPFM measurements reveal a significant surface potential increment for the P_{up} domain upon UV illumination (compare (c) with (i)).

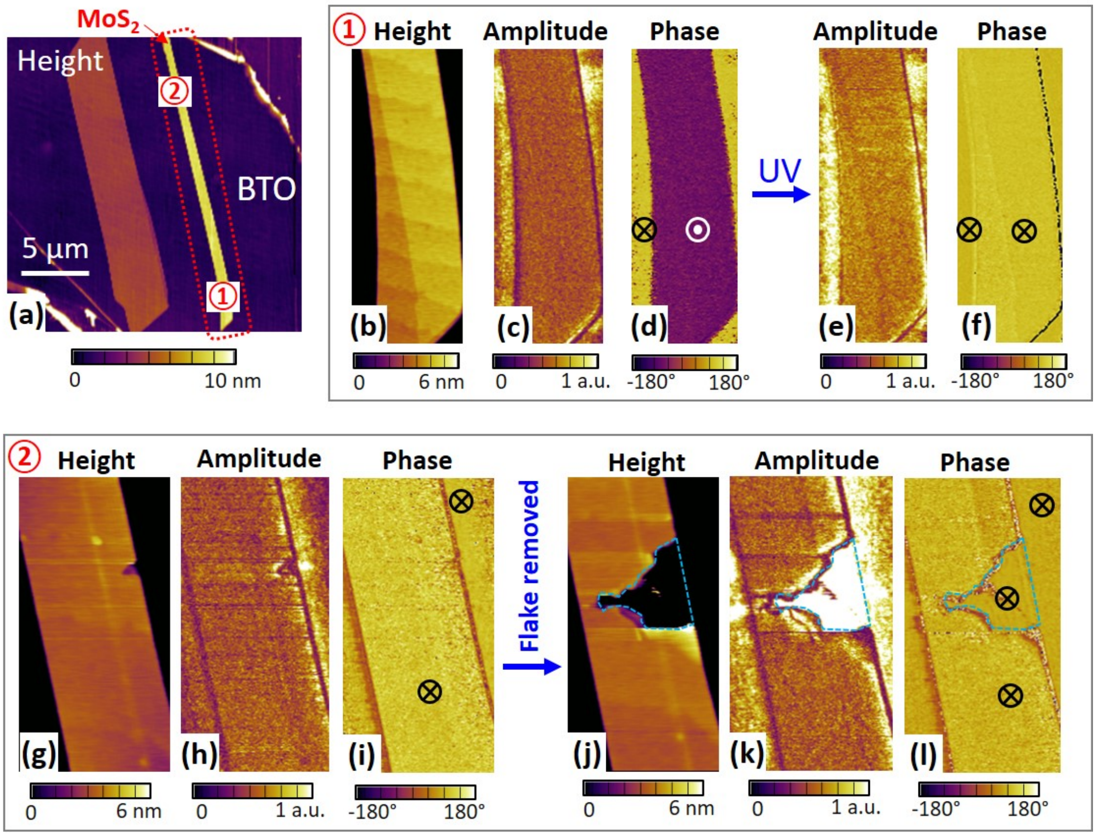

Supplementary Figure 3. Verification of optically-induced switching of $BaTiO_3$ under MoS_2. (a) Morphology of the whole MoS_2 flake that was tested (enclosed by red rectangle). (b-d) Morphology (b) and PFM (c, d) images of region 1 of MoS_2/BTO electrically switched to the upward polarization in the dark. Surrounding bare BTO surface is electrically switched to the downward polarization for reference. (e, f) PFM images of region 1 after the sample was illuminated by UV light for a few minutes. It can be seen that the BTO under MoS_2 was switched from the upward to the downward polarization state indicated by the same phase contrast of the MoS_2-covered and bare BTO film surface (f) and increased amplitude signal (e). To provide additional verification of the BTO polarization state under MoS_2, part of the MoS_2 flake in region 2 was mechanically removed by an AFM tip after UV illumination. (g-i) Morphology (g) and PFM images (h, i) of region 2 before removing part of the MoS_2 flake showing the downward polarization as in region 1. (j-l) Morphology (j) and PFM images (k, l) after part of the MoS_2 was removed (indicated by the blue line in (j)). Freshly exposed BTO surface shows a much stronger amplitude signal (k) than BTO still covered by MoS_2, and its phase contrast (l) indicates the downward polarization state, the same as for the bare BTO film. This observation

confirms that the PFM phase signal obtained through MoS_2 provides correct information about the polarization state of BTO, and that the UV illumination indeed switches the upward polarization to the downward state in the MoS_2/BTO heterostructures.

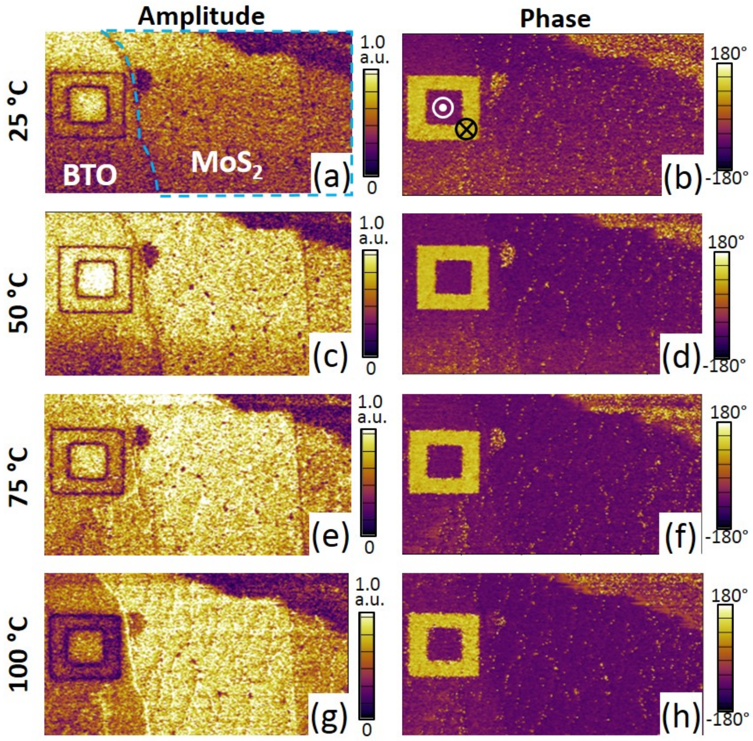

Supplementary Figure 4. Evaluation of domain retention at elevated temperatures. Initially, the MoS_2/$BaTiO_3$/$SrRuO_3$ junction is electrically poled to the upward polarization state at room temperature. The MoS_2 flake edge is marked by a blue dashed line in (a). For reference, square domains with upward and downward polarizations have been electrically written on the bare $BaTiO_3$ film surface before measurements. All images are acquired in the dark. It can be seen that the polarization states of both bare $BaTiO_3$ film and MoS_2/BTO/SRO heterostructure remain stable when sample is heated up to 100°C, except that the PFM amplitude signal of the $BaTiO_3$ film polarized downward is reduced. This result rules out a possibility of optically-induced polarization reversal being due to sample heating resulting from light absorption.

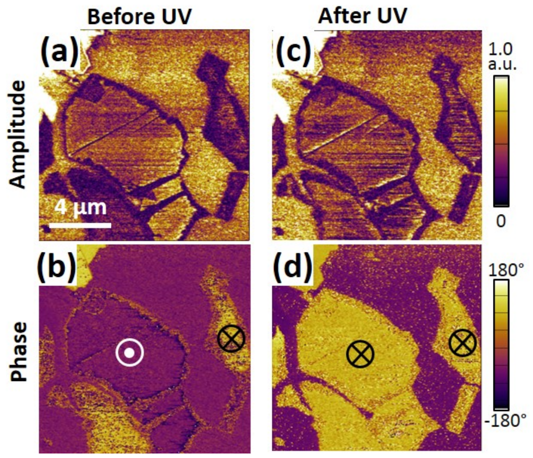

Supplementary Figure 5. Piezoresponse images of the same MoS_2 flakes shown in Figure 3. Comparison of the PFM images taken before (a,b) and after (c,d) illumination confirms optically-induced polarization reversal to the downward polarization state.

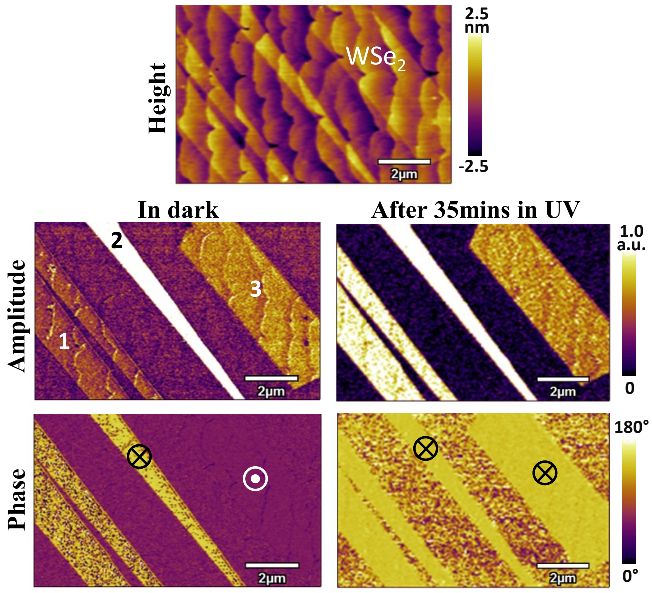

Supplementary Figure 6. Optical switching in the $WSe_2/BaTiO_3/SrRuO_3$ junctions. The UV light with the same intensity and wavelength was used to illuminate the WSe_2/BTO(12u.c.)/SRO junctions (WSe_2 thickness is about 1 nm). Flake 3, which was initially polarized upward, shows the downward polarization after UV illumination. There is no change in the downward polarization of flakes 1 and 2 due to UV illumination. The bare $BaTiO_3$ film has not been switched either. Thus, the observed behavior of the WSe_2/BTO/SRO junctions, which employ the p-type 2D semiconductor, is similar to that of the MoS_2/BTO/SRO junctions in that the optical switching occurs from the upward to the downward direction.

Supplementary Note 1: Computational Details

We investigated excitons in the presence of a perpendicular electric field of various MoS_2 slabs, in particular the trilayer-MoS_2 for which we show results in the article, by using Density Functional Theory (DFT) and many body perturbation theory (MBPT). We first obtain the ground state of trilayer MoS_2, i.e. the Kohn-Sham wavefunctions and eigenvalues by using DFT within the local density approximation (LDA) as implemented in the Quantum ESPRESSO (QE) suite [1]. The Mo 4s and 4p semi-core electrons are explicitly included as valence electrons. The plane-wave energy cutoff of 100 Ry and a 12 × 12 × 1 k-point grid are used for the ground state charge density. Spin-orbit coupling is taken into account by using spinorial wave functions and the optimized lattice parameter of 3.11 Å is used in the calculations. External electric fields were imposed using the standard saw-tooth-potential technique.

In order to cure the intrinsic band gap underestimation problem of the Kohn-Sham DFT, we perform GW [2,3] calculations using the YAMBO code [4]. In the GW step, the wave functions and energies in the G part (Green's function) or the dielectric function in the W part (screened Coulomb interaction) are not updated. This version of the GW method is called non-self-consistent GW and denoted as G_0W_0. In order to obtain the quasi-particle energies on a very fine k-point grid, we perform a G_0W_0 calculation on a scarce grid and fit a "scissor operator" for the difference between LDA energies and the quasi-particle energies for arbitrary values of k.

After obtaining the Kohn-Sham wavefunctions and quasi-particle energies, the optical spectra can be calculated by solving the Bethe-Salpeter equation [3,5,6] (BSE) in the following form

$$(E_{ck} - E_{vk})A^X_{cvk} + \sum_{c'v'k'} \langle cvk|K_{eh}|c'v'k'\rangle A^X_{c'v'k'} = \Omega^X A^X_{cvk} \quad (1)$$

where the electronic excitations are expressed in terms of electron-hole pairs ($|cvk\rangle$). The E_{ck} and E_{vk} are the conduction and the valence band energies of the vertical electronic transitions. K_{eh} is the interaction kernel which represents the screened Coulomb and the exchange interaction between electron and hole. (Dropping out this term leads would lead to excitations corresponding to independent electron hole pairs). The A^X_{cvk} and the Ω^X are the excitonic eigenvectors (expansion coefficients of excitonic wave functions) and the eigenvalues, respectively.

The wave function of the exciton can be expressed in terms of the Kohn-Sham orbitals and the expansion coefficients of the excitonic states as:

$$\psi^X(r_e, r_h) = \sum_{cvk} A^X_{cvk}\phi_{vk}(r_h)\,\phi_{ck}(r_e) \quad (2)$$

where $\phi_{ck}(r_e)$ and $\phi_{vk}(r_h)$ are the Kohn-Sham orbitals obtained from DFT calculations. The excitonic wave function contains six spatial coordinates corresponding to the position of electron (r_e) and hole (r_h). It is not possible to display these in a three-dimensional plot. Therefore, to visualize the charge density of excitons, we fix the hole in a likely position (taking into account the orbital character of the band, in which the hole resides) and plot the charge density of the electron as shown in Fig. 5(d) in the manuscript.

After obtaining all the ingredients, the imaginary part of the dielectric function is obtained as

$$\varepsilon_2(\hbar\omega) \sim \sum_X \left|\sum_{cvk} A^X_{cvk} \frac{\langle\phi_{ck}(r_e)|p_i|\phi_{vk}(r_h)\rangle}{(\epsilon_c - \epsilon_v)}\right|^2 \delta(\Omega^X - \hbar\omega - \Gamma) \quad (3)$$

Similar to G_0W_0 calculations, the BSE calculations are performed using the YAMBO code [4]. In our G_0W_0 and BSE calculations, we used a 30 × 30 × 1 k-point sampling and checked all the other parameters to get the converged absorption spectrum. In order to eliminate the artificial interaction between trilayer MoS_2 with its periodic image, we used a Coulomb cutoff along the z direction in our G_0W_0 and BSE calculations [7].

With the inclusion of the electric field, the symmetry operations are switched off in the calculations. This leads to more irreducible k-points (452 k-points) than in the case with symmetry (91 k-points) and makes the G_0W_0 calculation quite expensive. Therefore, we use the rigid ”scissor” operator obtained from the G_0W_0 calculation of trilayer-MoS_2 with symmetry (and without electric field). We note that in a recent calculation of excitons in bilayer MoS_2 under the influence of an electric field [9], the symmetry operations were not switched off. This leads to a strong overestimation of the change of the band-gap with respect to electric-field as demonstrated earlier in Ref [8].

Supplementary References

1. Giannozzi, P. et. al. Quantum espresso: a modular and open-source software project for quantum simulations of materials, *J. Phys.: Cond. Matt.* **21**, 395502 (2009).

2. Onida, G., Reining, L. and Rubio, A. Electronic excitations: density-functional versus many-body Green's-function approaches, *Rev. Mod. Phys.* **74**, 601-659 (2002).

3. Hedin, L. and Lundqvist, S. Effects of electron-electron and electron-phonon interactions on the one-electron states of solids, *Solid State Phys.* **23**, 1-181 (1970).

4. Marini, A., Hogan, C., Grüning, M. and Varsano, D. Yambo: an ab initio tool for excited state calculations, *Comput. Phys. Commun.* **180**, 1392-1403 (2009).

5. Strinati, G., Dynamical Shift and Broadening of Core Excitons in Semiconductors, *Phys. Rev. Lett.* **49**, 1519-1522 (1982).

6. Rohlfing, M. and Louie, S. G. Electron-hole excitations and optical spectra from first principles, *Phys. Rev. B* **62**, 4927-4944 (2000).

7. Azhikodan, D., Nautiyal, T., Shallcross, S. & Sharma, S. An anomalous interlayer exciton in MoS_2, *Sci. Rep.* **6**, 37075 (2016).

8. Liu, Q., Li, L., Li, Y., Gao, Z., Chen, Z., and Lu, J. Tuning electronic structure of bilayer MoS_2 by vertical electric field: a first-principles investigation, *J. Phys. Chem. C* **116**, 21556-21562 (2012).
